# The relationship between nociceptive brain activity, spinal reflex withdrawal and behaviour in newborn infants

**DOI:** 10.1038/srep12519

**Published:** 2015-07-31

**Authors:** Caroline Hartley, Sezgi Goksan, Ravi Poorun, Kelly Brotherhood, Gabriela Schmidt Mellado, Fiona Moultrie, Richard Rogers, Eleri Adams, Rebeccah Slater

**Affiliations:** 1 Department of Paediatrics, University of Oxford, OX3 9DU, UK; 2 Nuffield Department of Clinical Neurosciences, University of Oxford, OX3 9DU, UK; 3 Nuffield Department of Anaesthesia, John Radcliffe Hospital, OX3 9DU, UK.

## Abstract

Measuring infant pain is complicated by their inability to describe the experience. While nociceptive brain activity, reflex withdrawal and facial grimacing have been characterised, the relationship between these activity patterns has not been examined. As cortical and spinally mediated activity is developmentally regulated, it cannot be assumed that they are predictive of one another in the immature nervous system. Here, using a new experimental paradigm, we characterise the nociceptive-specific brain activity, spinal reflex withdrawal and behavioural activity following graded intensity noxious stimulation and clinical heel lancing in 30 term infants. We show that nociceptive-specific brain activity and nociceptive reflex withdrawal are graded with stimulus intensity (p < 0.001), significantly correlated (r = 0.53, p = 0.001) and elicited at an intensity that does not evoke changes in clinical pain scores (p = 0.55). The strong correlation between reflex withdrawal and nociceptive brain activity suggests that movement of the limb away from a noxious stimulus is a sensitive indication of nociceptive brain activity in term infants. This could underpin the development of new clinical pain assessment measures.

The measurement of infant pain is difficult due to the inability of an infant to describe the experience verbally. Consequently, numerous surrogate measures of pain, such as change in facial expression, heart rate and nociceptive-specific brain activity, have been used in an attempt to quantify pain intensity^1^,^2^,^3^, and guide pain management and treatment^4^. However, interpreting these measures is difficult as there is discordance between the noxious-evoked behavioural, physiological and brain activity^5^,^6^,^7^. For example, lack of concordance between facial expression and change in heart rate has been reported^5^, and nociceptive brain activity can be recorded without concomitant changes in facial expression^7^. Moreover, sucrose (an intervention thought to alleviate pain) can reduce clinical pain scores without altering nociceptive brain and spinal cord activity^8^. To overcome these difficulties multidimensional pain assessment tools are recommended for use in the clinical setting^4^. However, establishing the most clinically appropriate surrogate measures of infant pain has not been grounded by our knowledge of nociceptive processing in the immature infant central nervous system (CNS).

In animal studies, one of the most commonly used surrogate measures of pain is nociceptive reflex withdrawal, and consequently it has underpinned much of our mechanistic understanding of nociceptive processing^9^,^10^. In adults, the magnitude of the nociceptive reflex withdrawal is highly correlated with perceived pain intensity^11^. Nociceptive reflex withdrawal has also been characterised in infants and is also correlated with stimulus intensity^12^, but has longer duration, higher amplitude and is evoked at a lower threshold compared with adults^13^. However, it is not known how nociceptive reflex withdrawal activity relates to nociceptive processing in the infant brain. Understanding this relationship is important because spinally and cortically mediated nociceptive responses are developmentally regulated and may not necessarily be predictive of one another in the immature nervous system^13^,^14^,^15^. This is also of interest from a clinical perspective as protective reflex withdrawal responses can be visually observed, and limb and body movements have been incorporated into some clinical pain assessment tools^16^,^17^,^18^.

To date recordings of electrophysiological nociceptive brain activity in infants have been made following clinically essential procedures, allowing limited scope to characterise the patterns of activity^19^,^20^,^21^. In this study, we investigate whether low intensity experimental noxious stimulation evokes nociceptive-specific patterns of brain activity and spinally mediated reflex withdrawal. The aims were to establish whether patterns of reflex withdrawal activity and nociceptive brain activity encode stimulus intensity, and whether the magnitude of the nociceptive brain activity is related to the magnitude of the evoked reflex withdrawal in a population of term infants.

## Results

### Characterisation of nociceptive-specific brain activity in newborn infants during a clinically required heel lance

**Figure 1:**
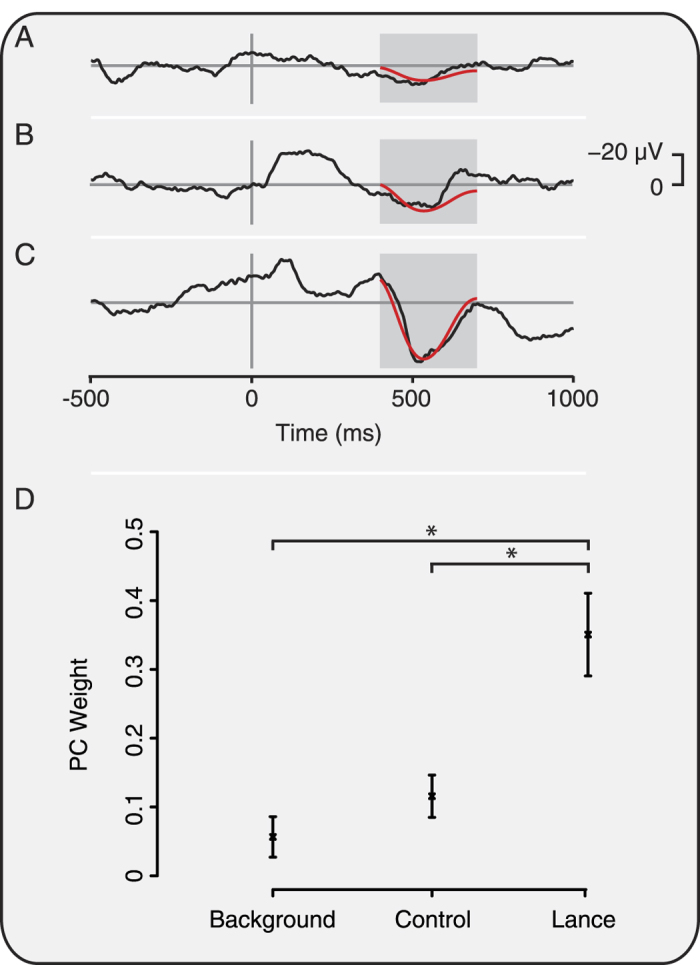
Characterisation of nociceptive-specific brain activity. Average EEG activity recorded during (**A**) background, (**B**) in response to non-noxious control stimulation, and (**C**) following noxious heel lance in 6 infants. The nociceptive-specific principal component (PC) is overlaid in red. The PC weights were significantly greater (*p < 0.05) in response to lance than background and control (**D**). Error bars indicate standard error of the mean.

To determine whether nociceptive-specific brain activity was evoked in response to experimental noxious stimuli, the activity was first defined in response to a known noxious stimulus – a clinically required heel lance. EEG activity was recorded during a background period and following non-noxious control stimulation and noxious heel lance. Consistent with previous reports, nociceptive-specific activity was identified using Principal Component Analysis (PCA)^14^,^21^,^22^. The second principal component (PC), which accounted for 42.5% of the variance, was defined as nociceptive-specific because the weight of this component was significantly greater in response to the noxious heel lance, compared with non-noxious control stimulation and background EEG activity (n = 6; p = 0.0026, Fig. 1).

### Experimental noxious stimulation did not cause a significant increase in clinical pain scores

Premature Infant Pain Profile (PIPP) scores were calculated in a background period (prior to stimulation), and after the first and last experimental noxious stimuli in a train of 10 stimuli at a force of 128 mN in 10 infants. The mean PIPP score was 2.8 ± 1.6 (mean ± standard deviation) during a background period; 3.9 ± 3.4 after the first experimental noxious stimulus; and 3.7 ± 2.0 after the last stimulus. There was no significant difference between PIPP scores across the 3 stimulus conditions (p = 0.55). The total score from the 3 facial expression components was also low (background period: 0.2 ± 0.4, first experimental noxious stimulus: 1.6 ± 2.9, and last experimental noxious stimulus: 0.7 ± 2.0) and was not significantly different across stimulus conditions (p = 0.56).

### Experimental noxious stimulation evokes a nociceptive-specific pattern of brain activity

**Figure 2:**
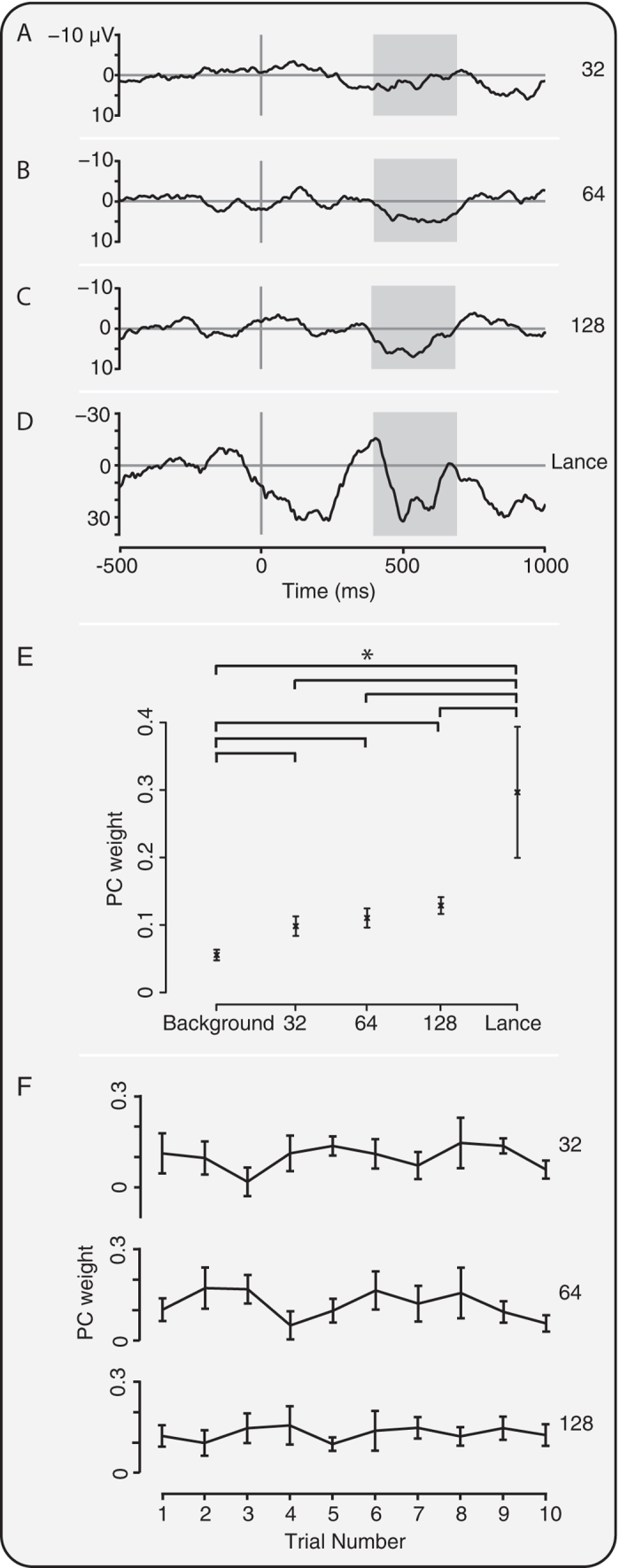
Experimental noxious stimuli evoke nociceptive-specific brain activity. Average traces (without Woody filtering jitter) in response to the (**A**) 32 mN, (**B**) 64 mN, and (**C**) 128 mN experimental noxious stimulus, and (**D**) clinically required heel lance. (**E**) PC weight of the nociceptive-specific brain activity was graded with intensity and was significantly different to background data following all the experimental noxious stimuli and heel lance (*p < 0.05). (**F**) PC weight did not significantly change with stimulus number for any of the experimental noxious stimuli. Error bars indicate standard error of the mean. Grey boxes indicate the time window 400–700 ms.

Having characterised the pattern of nociceptive-specific brain activity following clinical heel lance in a sample of infants (Fig. 1), we then determined whether this activity was evoked by the experimental noxious stimuli (n=12), and for comparison, by a clinical heel lance performed in an independent sample of infants (n=4). The heel lance and the experimental stimuli (with forces of 32 mN, 64 mN and 128 mN) evoked nociceptive-specific brain activity that was significantly greater than activity recorded in the background period (p < 0.001, one-way ANOVA; post-hoc comparison of background and 32 mN stimuli: p = 0.040; 64 mN stimuli: p = 0.007; 128 mN stimuli: p = 0.0002; heel lance p = 0.0005, Fig. 2). The nociceptive-specific activity was significantly higher in response to the heel lance than any of the experimental noxious stimuli (p < 0.03). Furthermore, the magnitude of the nociceptive-specific brain activity increased with increasing stimulus intensity (p < 0.001), and was not dependent on stimulus number (p > 0.05, see Fig. 2F).

### Experimental noxious stimulation evokes nociceptive reflex withdrawal activity that is correlated with nociceptive-specific brain activity

**Figure 3:**
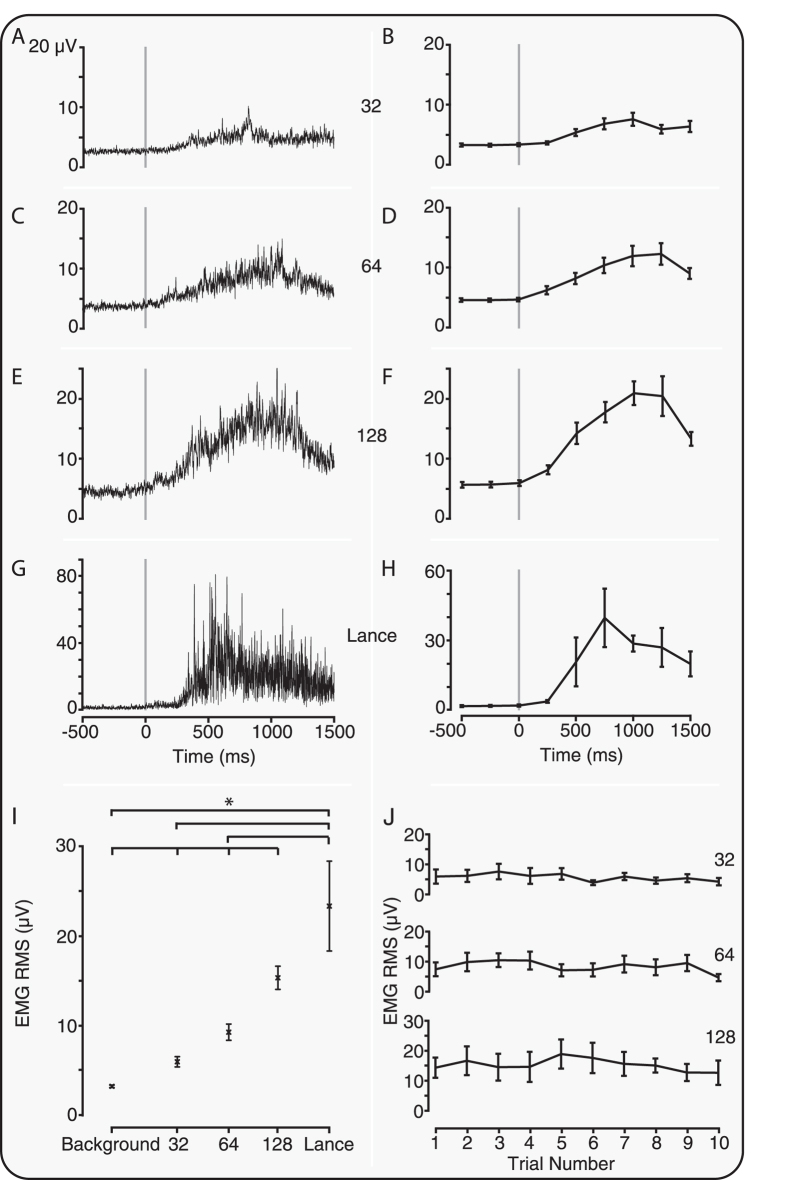
Experimental noxious stimuli evoke significant reflex withdrawal. Average EMG response (**A**,**C**,**E**,**G**) and average RMS (**B**,**D**,**F**,**H**) to the (**A**,**B**) 32 mN, (**C**,**D**) 64 mN, and (**E**,**F**) 128 mN experimental noxious stimulus and (**G**,**H**) clinically required heel lance. (**I**) The reflex withdrawal was graded with stimulus intensity and for all stimuli the response was significantly different to background activity (*p < 0.05). (**J**) The reflex withdrawal response was not significantly different across stimulus number for any of the experimental noxious stimuli. Error bars indicate standard error of the mean.

**Figure 4:**
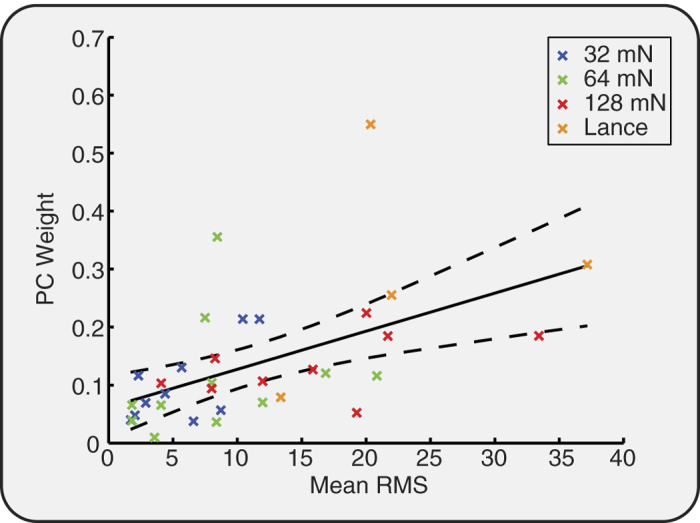
Reflex withdrawal and nociceptive-specific evoked brain activity is correlated. PC weight of the nociceptive-specific brain activity plotted against average EMG RMS for each infant and each stimulus. The regression line (solid) and 95% confidence intervals (dashed) are shown in black.

The experimental noxious stimulation and clinical heel lance evoked a reflex withdrawal of the stimulated leg that could be quantified using surface EMG electrodes. Consistent with the evoked brain activity, the clinical heel lance and the experimental noxious stimuli evoked reflex withdrawal of the stimulated leg that was significantly greater than activity recorded in the background period (p < 0.001, Kruskal-Wallis test; post-hoc comparison of background and 32 mN: p = 0.01; 64 mN, 128 mN and heel lance: p < 0.005, Fig. 3). The magnitude of the reflex withdrawal also increased with increasing stimulus intensity (p < 0.001) and was not dependent on stimulus number (p > 0.05, Fig. 3J). In addition, the magnitude of the nociceptive-specific brain activity was significantly correlated with the magnitude of the reflex withdrawal (r = 0.53, p = 0.001, see Fig. 4).

If observations of limb withdrawal are to be useful in a clinical setting then quantification of the EMG activity should be reflected in visible movement. Visible reflex withdrawal was observed in 56.1% of trials, and was dependent on stimulus intensity (visible withdrawal: 32 mN: 32.6%; 64 mN: 48.0% and 128 mN: 72.5%). As expected, the magnitude of the reflex withdrawal was significantly greater in the trials where limb movement was observed compared with those without visible movement (p < 0.001; RMS (without visible movement) = 5.0 ± 0.4 μV, mean ± standard error of the mean; RMS (with visible movement) = 13.1 ± 0.88 μV).

### Bilateral reflex withdrawal is observed at greater stimulus intensities

**Figure 5:**
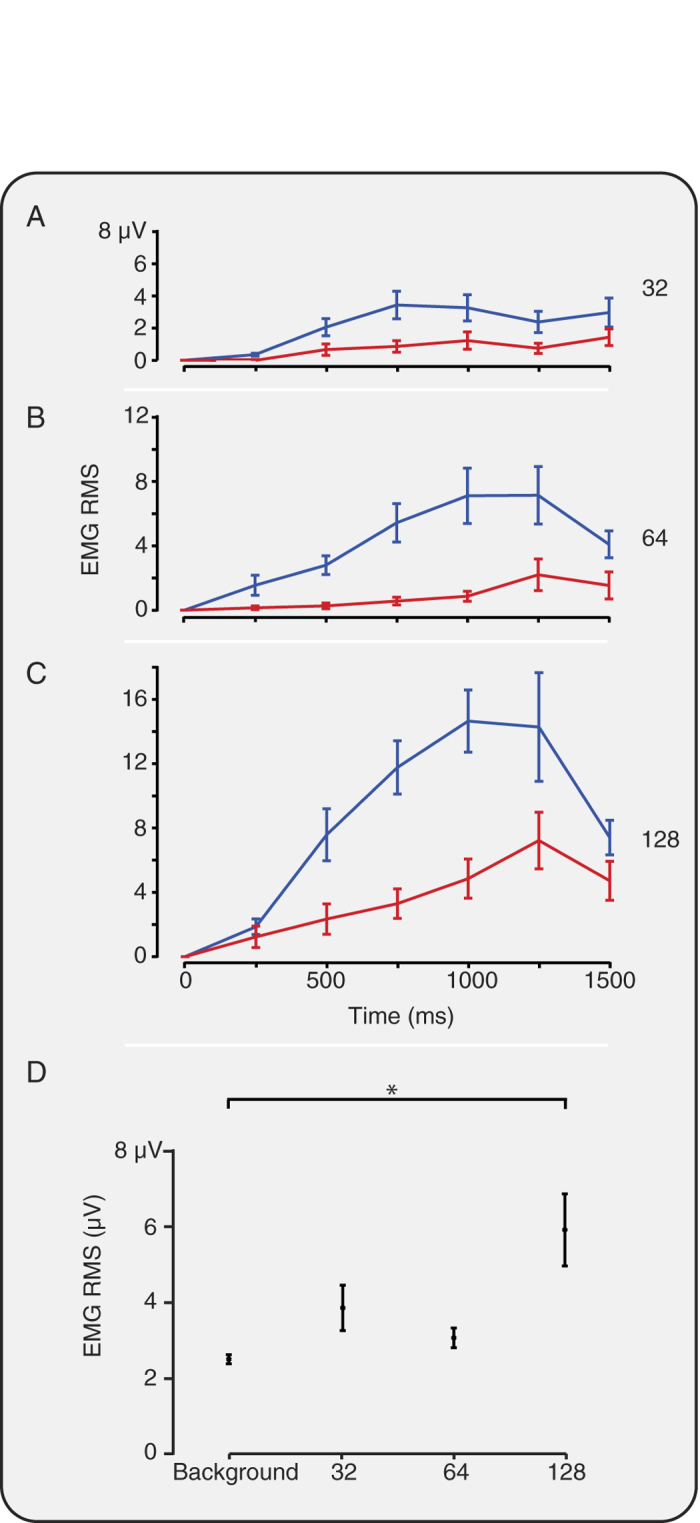
Bilateral leg withdrawal occurs with higher intensity noxious stimuli. EMG RMS in response to the (A) 32 mN, (B) 64 mN, and (C) 128 mN experimental noxious stimulus for the ipsilateral, i.e. stimulated, (blue) and contralateral (red) leg. (D) The average EMG RMS in the contralateral limb in response to stimuli (in the subset of trials when significant ipsilateral reflex withdrawal occurred) and in background activity. Only following the 128 mN stimuli was the response significantly different to background activity (*p < 0.05). Error bars indicate standard error of the mean.

Reflex withdrawal of the contralateral limb was also observed, but had lower magnitude than the activity evoked in the ipsilateral limb (Fig. 5). When considering the subset of trials where significant ipsilateral reflex withdrawal was defined to have occurred, significant reflex withdrawal of the contralateral limb was evoked by the 128 mN stimulus (p = 0.003, Fig. 5). However, when lower intensity stimulation was applied at a force of 32 mN and 64 mN, the magnitude of the contralateral limb reflex withdrawal was not significantly different from background activity (p > 0.05, Fig. 5). Therefore, at lower stimulus intensities reflex withdrawal is evoked in the ipsilateral limb, whereas at higher stimulus intensities bilateral reflex withdrawal is observed.

## Discussion

We have characterised the pattern of nociceptive brain and spinal cord activity evoked by acute experimental noxious stimulation in a sample of term infants. We demonstrate that in infants the stimuli evoke nociceptive-specific brain activity that increases with stimulus intensity, and is strongly correlated with spinally mediated reflex leg withdrawal. Furthermore, the extent to which the reflex withdrawal is bilaterally mediated is dependent on stimulus intensity.

The stimuli applied in this study are likely to predominantly activate Aδ-fibre nociceptors^23^,^24^. The application of the same experimental noxious stimuli in adults are verbally described as eliciting a ‘sharp’ and ‘pricking’ sensation using the McGill pain questionnaire^25^ and evoke event-related potentials (when applied with a force of 128 mN) in recorded EEG^24^. We show that in infants, the 32 mN, 64 mN and 128 mN force evokes a nociceptive-specific pattern of brain activity, that is comparable to that evoked by clinical heel lance but with significantly smaller magnitude. There is some variability in the magnitude of the evoked responses between infants, which could relate to genetics, environment, or previous pain exposure. Understanding the factors that contribute to this observation would require further investigation.

Responses to the experimental noxious stimuli were identified by comparing the pattern of nociceptive brain activity with that evoked by a clinical heel lance. To account for latency variation between evoked responses, some latency jitter (±50 ms) was allowed between trials prior to identifying nociceptive-specific activity. Latency variation could arise due to individual infant differences or due to the accuracy in which the precise timing of the stimuli can be determined. It is possible that latency jitter may result in the alignment of waveforms recorded in background EEG activity, even in absence of evoked nociceptive brain activity. However, as latency jitter was allowed in both the background activity and responses to noxious stimuli, and significant differences were observed, this provides a robust approach.

In this study, neither sensitisation nor habituation were observed when the infants were re-presented with the experimental noxious stimuli, however, this does not exclude the possibility that these phenomena would occur if the stimuli were presented at different intensities, inter-stimulus intervals, over a longer duration, or to preterm infants, as have been observed in other studies^12^,^13^,^26^,^27^. Furthermore, responses to noxious stimuli may be dependent on prior experience of the infant^22^,^28^, which may be particularly relevant when considering prematurely-born infants or those receiving neonatal care, who are often exposed to multiple invasive clinically required procedures^29^. Such prior experience may not only alter brain responses to individual noxious stimuli^22^, but may also influence whether repeated exposure to noxious stimuli induces sensitisation or habituation. More detailed studies in this area may be relevant when considering clinical practices such as clustered care, where a number of essential clinical procedures are carried out in a short time window to limit overall infant handling^30^.

The magnitude of both the nociceptive-specific brain activity and spinal reflex withdrawal activity increased with increasing stimulus intensity, and were strongly correlated. This is consistent with adult studies where nociceptive reflex withdrawal and noxious-evoked brain activity increases with stimulus intensity, and is correlated with subjective pain report^11^,^31^,^32^. However, in contrast to adults, infant reflexes are longer duration and can be elicited by lower intensity non-noxious stimulation^13^.

Rat pups also have a low flexion reflex threshold compared to mature adults, and a postnatal decline in reflex amplitude and duration has been demonstrated^27^,^33^. This pattern of development likely reflects maturation of descending inhibitory control^34^ and reorganisation of the primary afferent input in the spinal dorsal horn^35^,^36^. It has been suggested that the high sensitivity of infant reflexes - which can be elicited by tactile stimulation – reflects the need for sensory input at a critical stage of development^13^,^37^. We demonstrate that term infants have the ability to encode noxious stimulus intensity, and while infant reflexes are more sensitive than adults, the processing of nociceptive information in both the brain and spinal cord is sensitive to stimulus intensity. In a comparable infant fMRI study investigating nociceptive processing, the trend for intensity encoding in many brain structures was evident, although not statistically significant in individual brain regions^25^. In this EEG study we have not localised the source of the nociceptive-specific brain activity, as a relatively low number of recording electrodes were applied. However, it is likely that a number of brain regions contribute to the patterns of activity observed here.

The laterality of reflexes is developmentally regulated. von Frey hair stimulation of the abdomen evokes an abdominal reflex and bilateral hip flexion in infants before 36 weeks gestation, after which only unilateral hip flexion occurs^38^. In term infants, bilateral limb withdrawal is observed in response to noxious clinical heel lancing^13^. Here, we demonstrate that significant reflex withdrawal of the contralateral limb occurs with a stimulus intensity of 128 mN, whereas stimulation with a force of 32 mN and 64 mN only elicits significant reflex withdrawal in the ipsilateral limb. Together this data suggests that bilateral reflex withdrawal is more likely to occur following higher intensity noxious stimulation in term infants. Reflexes are intrinsic protective behaviours that have evolved to minimise tissue damage and protect the body from harm. These results suggest that infants produce energy-efficient reflex responses. More vigorous bilateral responses are elicited in response to high intensity noxious stimulation, providing a greater likelihood of withdrawing from an offending stimulus.

The Premature Infant Pain Profile (PIPP) is a composite multimodal measure, incorporating measures of heart rate, oxygen saturation and facial expression change, and has been well validated for acute pain^39^. The experimental noxious stimuli used in this study did not evoke a significant change in PIPP scores in term infants. Furthermore, the magnitude of the reflex withdrawal and nociceptive-specific brain activity, was substantially lower than that evoked by clinical heel lance. As the experimental noxious stimulation can be repeated in the same infant multiple times, without causing behavioural discomfort or clinical concern, it provides an opportunity to develop a more detailed mechanistic understanding of nociceptive processing in the immature central nervous system. This has the potential to transform the study of infant pain. For example, these techniques could be used to evaluate the anti-nociceptive properties of analgesics in infants - a greatly understudied but crucial area of research. Nevertheless, to date the experimental noxious stimulators have not been tested in premature infants and their suitability in other clinical populations needs to be established.

The assessment of pain in infants is complex, and in non-verbal populations it is ultimately not possible to know whether a person is in pain. It has recently been demonstrated that brain regions that encode the sensory and affective components of adult pain, including the anterior cingulate, insula and somatosensory cortices are also active in infants^25^. As cortical processing is necessary for the emotional and conscious perception of pain, examining nociceptive-specific brain activity is likely to provide the best surrogate measure^40^.

In this study, we demonstrated that reflex withdrawal is highly correlated with nociceptive brain activity, sensitive to stimulus intensity, and can be recorded in the absence of facial expression change. Although reflex withdrawal is criticised as a pain assessment measure due to its lack of nociceptive specificity^17^, this is also true for facial expression and autonomic responses^41^. As the experimental stimuli used here were applied at an intensity that does not evoke significant changes in PIPP scores, the results suggest that the PIPP score is not sensitive enough to detect responses to low intensity noxious stimulation. The importance of considering infant body movements in the absence of facial expression change has been previously suggested^18^, and a number of pain assessment tools already consider measures of infant body movement^16^,^17^,^42^,^43^. Given the high proportion of infants that do not mount facial expression responses following noxious stimulation^28^,^44^, the clinical importance is clear. Moreover, the observation that bilateral limb withdrawal is more likely to reflect greater nociceptive signalling means this could be an additional factor to consider during clinical pain assessment. Nevertheless, visual clinical observations of limb withdrawal could be limited if limbs are restricted during invasive procedures or covered by clothing or blankets.

This study supports previous research that shows that infants can process nociceptive information at the cortical level without manifesting a change in facial expression^7^. This has important consequences for the management and treatment of infant pain, as nociceptive information transmitted to the cortex may not be reflected when observing changes in facial expression, or recorded using current clinical pain assessment tools. In the absence of verbal report, decisions about the treatment and management of infant pain should be based on a comprehensive understanding of the relationship between brain, spinal and behaviourally-mediated responses to noxious stimulation. This data provides a clear neurobiological rationale that may be used to underpin the development and validation of new clinical pain assessment measures.

## Methods

### Subjects

30 infants were recruited from the Maternity Unit and Special Care Baby Unit at the John Radcliffe Hospital between May 2012 and January 2015. Infants were 37 to 42 weeks gestation at the time of study and less than 10 postnatal days. Medical charts were reviewed and, at the time of study, infants were assessed as clinically stable. Infants were not eligible for inclusion in the study if they had previous surgery, had intraventricular haemorrhage or periventricular leukomalacia, were born with congenital malformations or other genetic disorders, or were currently requiring respiratory support or analgesic medication. Ethical approval (National Research Ethics Service, REC reference: 12/SC/0447 & 11/LO/0350) and informed written parental consent was obtained prior to each study. The study was carried out in accordance with the standards set by the Declaration of Helsinki and Good Clinical Practice guidelines.

### Electrophysiological recordings (EEG and EMG recordings)

Electrophysiological activity was acquired with the SynAmps RT 64-channel headbox and amplifiers (Compumedics Neuroscan), with a bandwidth from DC-400 Hz and a sampling rate of 2 kHz. CURRYscan7 neuroimaging suite (Compumedics Neuroscan) was used to record the activity. All equipment conformed to the electrical safety standard for medical devices, IEC 60601-1.

EEG was recorded at eight scalp electrodes (Ambu Neuroline disposable Ag/AgCl cup electrodes) in positions Cz, CPz, C3, C4, FCz, Oz, T3 and T4 according to the modified international 10–20 system. The reference electrode was positioned at Fz and the ground was placed on the forehead. EEG conductive paste (Elefix EEG paste, Nihon Kohden) was used to optimise contact with the scalp. Impedances were reduced by gently rubbing the skin with EEG preparation gel (NuPrep gel, D.O. Weaver and Co.) prior to electrode placement. In 3 studies a reduced electrode montage was used, but activity was always recorded at the Cz electrode site. In 3 other studies the reference electrode was placed at Fpz and re-referenced to Fz post-acquisition.

Bipolar EMG electrodes (Ambu Neuroline 700 solid gel surface electrodes) were placed on the biceps femoris of both legs.

### Experimental procedures

#### Clinical heel lancing

Heel lancing was performed as part of the infants’ routine clinical care (n = 10). EMG and EEG activity was recorded during a period of background, a control lance (where the lancet was rotated by 90 degrees and held against the infant’s foot so that the blade did not contact the infant’s heel when released), and a clinically required heel lance. The lance and control lance were time-locked to the EEG and EMG recordings using an event-detection interface and accelerometer^3^. Where this was not possible, the events were time-locked using a microphone held against the lancet, with the audio recording directly linked to the electrophysiological recordings (n = 3).

#### Experimental noxious stimulation

Non-tissue damaging acute experimental noxious stimuli (PinPrick, MRC systems) were applied to the left heel in 12 infants (2 of these infants also had a heel lance). Three stimulus intensities were applied in a randomised order (applied force: 32 mN (n = 11 infants); 64 mN (n = 12 infants); and 128 mN (n = 9 infants)). At each stimulus intensity a train of 9.8 ± 2.1 stimuli (mean ± standard deviation) were applied with an inter-stimulus interval of 18.8 ± 14.1 seconds. Throughout the study period the infant’s foot was loosely held by the experimenter so that the reflex of the foot was not impeded. Prior to giving consent, parents were shown the experimental stimulators and were able to test the stimulators on themselves. In nearly all cases the parents chose to remain with their infants during the studies and in no case was consent withdrawn.

**Figure 6:**
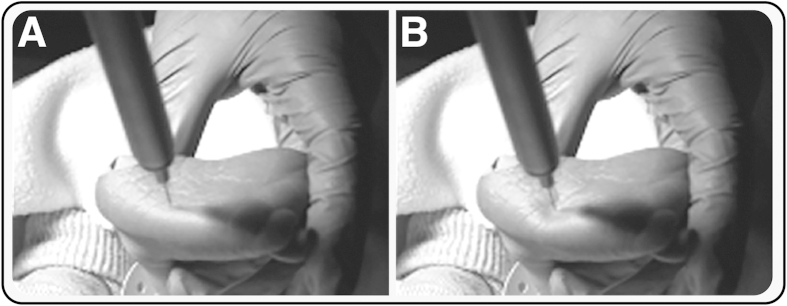
Time-locking of experimental noxious stimuli. Experimental noxious stimuli were time-locked to EEG and EMG recordings using a high-speed video camera. Example images of (**A**) the point of first contact of the stimulus with the skin, and (**B**) the point at which the barrel of the stimulus was first depressed (i.e. the point at which the force was first applied), which was taken as the point of stimulation. We would like to acknowledge Ravi Poorun for taking the photographs and for preparing the images.

The experimental stimuli were time-locked to the EEG and EMG recordings using a high-speed camera (Firefly MV, Point Grey Research Inc.) that was directly linked to the recordings at the time of acquisition^45^. The video recordings were reviewed post-acquisition and the time of stimulation was manually event-marked at the point where the barrel of the stimulator was first depressed, i.e. the time at which the force is first applied (Fig. 6).

#### Clinical Pain Scores

Clinical pain scores were calculated in response to experimental noxious stimuli in an independent sample of infants (n = 10) using the Premature Infant Pain Profile (PIPP)^39^. The PIPP score was calculated in a background period before any stimuli were applied, and following the first and last experimental stimuli. Ten experimental noxious stimuli (force = 128 mN) were applied to the infant’s left heel with a minimum inter-stimulus interval of 10 seconds, while the foot was gently supported by the experimenter. The inter-stimulus interval was extended to include 15 seconds before and 30 seconds after the 1^st^ and 10^th^ stimuli, which enabled the PIPP scores to be calculated following a single noxious event. In one infant, only 5 stimuli were applied as the infant became restless during the study.

### Data analysis

#### EEG analysis

EEG was filtered 0.5–70 Hz (or when used in principal component analysis – see below – it was filtered 0.5–8 Hz), with a notch filter at 50 Hz. 1500 ms epochs were extracted with 500 ms before the stimulus and traces were baseline corrected to the pre-stimulus mean. EEG data epochs were rejected if gross movement artefacts were present.

Nociceptive-specific brain activity was defined at the Cz electrode by comparison of background EEG, and responses to the control heel lance and heel lance (n = 6 infants). Data from these conditions was aligned in the time window 400–700 ms using Woody filtering with a maximum shift of −50 to +100 ms (aligning to the average of the data). Principal Component Analysis (PCA) in the region 400–700 ms was then used to identify the Principal Components (PCs) of activity in the background, control and lance data^21^. The nociceptive-specific component was identified as the PC with significantly different weights in the lance condition compared with background and control data. The first two PCs accounted for 96% of the variance in the data so only these components were considered.

To calculate the corresponding weights of the nociceptive-specific component in the experimental noxious data, the nociceptive-specific PC was projected onto the experimental noxious responses, making use of singular value decomposition^14^. Let *X*_*0*_ be the original data set (in this case the heel lance, control heel lance and background EEG data from the independent sample of infants). The singular value decomposition of *X*_*0*_ is given by:
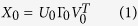


Given a new data set, *X*_*1*_ (in this case the EEG responses to the experimental noxious stimuli) the corresponding weights, U_1_, of the associated PCs of *X*_*0*_ are given by:
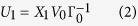


Using this approach we calculated the weights of the nociceptive-specific component for the experimental noxious data in the region 400–700 ms after the stimulus at the Cz electrode. This weight was calculated for each individual EEG trace, which was first Woody filtered, with a maximum jitter of ± 50 ms, in the region of 400–700 ms after the stimulus by identifying the maximum correlation with the nociceptive-specific component. The same Woody filtering approach was also applied to background data. When calculating the correlation between EMG and EEG responses, the average response for each infant was used to calculate the PC weight.

The nociceptive-specific component was also projected onto the responses to heel lance in an independent population of infants (n = 4) (i.e. these infants were not included in the original identification of the nociceptive-specific component). This provided a valid approach whereby the weights of the nociceptive-specific activity evoked by the heel lance could be statistically compared with the weights of the nociceptive-specific activity evoked by the experimental noxious stimulation.

#### EMG analysis

EMG signals were filtered between 10–500 Hz with a notch filter at 50 Hz (and harmonics). Epochs were extracted from 500 ms before to 1500 ms after the stimulus and rectified. The data was divided into 250 ms windows and in each window the root mean square (RMS) signal was calculated. The post stimulus mean was calculated as the mean across the first four windows after the stimulus (0–1000 ms) and the pre-stimulus mean was calculated across the two windows prior to the stimulus^8^.

Data was excluded (for example, due to movement artefact) according to the following definitions: post-stimulus mean > population mean plus twice the standard deviation; pre-stimulus mean > population mean plus twice the standard deviation; or the fold increase (post-stimulus mean divided by the pre-stimulus mean) > population mean plus twice the standard deviation.

To graphically compare the contralateral and ipsilateral reflex withdrawal the RMS responses were baseline corrected (Fig. 5), which accounted for differences in the RMS EMG activity between the limbs. To ascertain whether the observed reflexes were bilateral, the magnitude of contralateral reflex withdrawal was considered when significant reflex withdrawal occurred in the ipsilateral leg. Ipsilateral reflex withdrawal was defined in individual trials as significant when the average post stimulus RMS activity was greater than a threshold. The threshold was defined as 95% of the distribution of the background average RMS activity. Visible movement of the ipsilateral limb in response to the stimulus was assessed post-acquisition by reviewing videos of infant foot movement.

#### PIPP scores

A video camera was used to record the infant’s facial expression. An LED was synchronised to flash when the experimenter pressed a foot pedal at the point of stimulation. A pulse oximeter probe (OxiMax N-600 pulse oximetry monitor, Nellcor) was placed on the infant’s right foot to record oxygen saturation and heart rate. The data were downloaded to an external PC, or, if this was not possible, manually read from the monitor at the time of study. Change in heart rate and oxygen saturation scores were calculated according to the PIPP score^39^.

**Figure 7:**
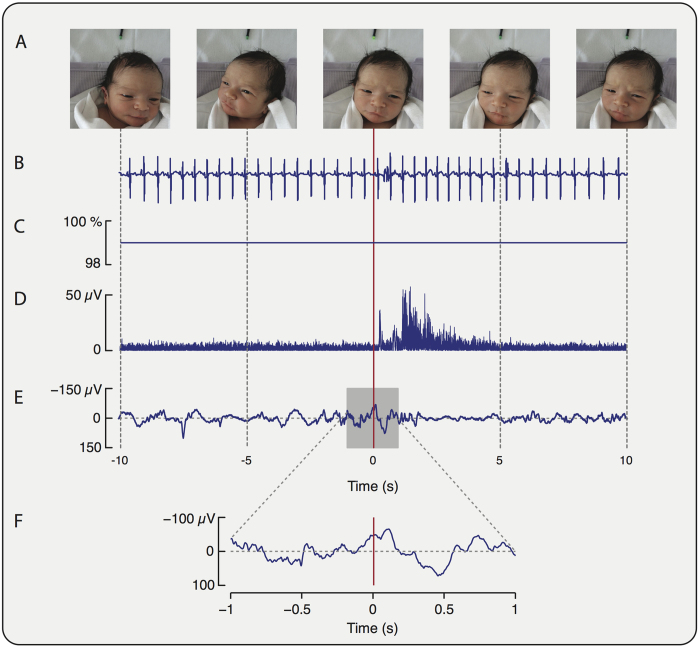
Behavioural, physiological, spinal cord and brain activity recorded in response to an experimental noxious stimulus. Recordings of (**A**) facial expression; (**B**) heart rate; (**C**) oxygen saturation; (**D**) EMG activity recorded from the biceps femoris of the stimulated leg and (**E**,**F**) EEG activity at the Cz electrode site are shown during application of a 128 mN stimulus. (The stimuli was applied at time = 0 seconds, and the facial expression screen shots are shown at −10, −5, 0, 5 and 10 seconds).

Post-acquisition, two trained investigators (who were blinded to the stimulus condition) reviewed the video recordings independently and calculated the PIPP score^39^. Behavioural state and facial expression (nasolabial furrow, eye squeeze and brow bulge) were independently assessed from the video recordings. Figure 7 shows example recordings of facial expression, heart rate, oxygen saturation, spinal reflex withdrawal activity and nociceptive brain activity in response to the experimental noxious stimulus.

### Statistical analysis

Comparison of PC weights in response to the heel lance, control lance and in the background EEG was calculated using a repeated-measures ANOVA. Intra-rater and inter-rater reliability of PIPP scores was calculated using intra-class correlation. The intra-rater reliability was 0.96, and the inter-rater reliability was 0.84. Comparison of PIPP scores between background, first experimental noxious and last experimental noxious stimuli was carried out using a repeated measures ANOVA. Sphericity was checked using Mauchly’s Test for Sphericity. A one-way ANOVA was used to compare the nociceptive-specific brain activity evoked by the heel lance, experimental noxious stimulation and background activity. As the data was not normally distributed, a Kruskal-Wallis test was used to compare the EMG RMS responses following the same stimulus conditions (for both the contralateral and ipsilateral leg).

Linear regression, with subject taken as a factor, was used to calculate the relationship between reflex withdrawal and stimulus intensity, and the relationship between the nociceptive-specific brain activity and stimulus intensity (across responses to 32, 64 and 128 mN experimental noxious stimuli and background activity). Linear regression was also used to calculate the correlation between nociceptive-specific brain activity and reflex withdrawal response, and Pearson’s linear correlation coefficient was calculated. The relationship between the stimulus number and evoked responses (at each stimulus intensity) was investigated using a Friedman’s test (due to the relatively low subject numbers in comparison with the number of trials). Where a single missing trial occurred, the missing value was included by regression substitution. Subjects with more than one missing trial (due to artefact rejection or where the camera angle did not allow the onset of the stimulus to be determined) were excluded from this analysis. Comparison of the EMG RMS between trials with and without visible movement was carried out using a Mann-Whitney U test. When necessary, post-hoc comparisons were conducted using t-tests and Mann-Whitney U tests with Holm’s method used to correct for multiple comparisons.

## Additional Information

**How to cite this article**: Hartley, C. *et al*. The relationship between nociceptive brain activity, spinal reflex withdrawal and behaviour in newborn infants. *Sci. Rep*. **5**, 12519; doi: 10.1038/srep12519 (2015).
